# The impact of clinical audits on improving the effectiveness of type 2 diabetes mellitus (T2DM) CARE in primary health centers: A comprehensive pre-post analysis through multi-layered intervention [ICAE-DM CARE STUDY PROTOCOL]

**DOI:** 10.1371/journal.pone.0313664

**Published:** 2025-07-15

**Authors:** Ansif Pallath Majeed, Niyaz Panakaje, Shajitha Thekke Veettil, Kiran Harikumar, Hanan Al Mujalli, Abdul Ali Shah, Noora Jassim AlKubaisi, Ahmed Sameer Alnuaimi

**Affiliations:** 1 Yenepoya (Deemed to be University), Mangalore, India; 2 Primary Health Care Corporation, Doha, Qatar; 3 Yenepoya Research Centre for Finance and Entrepreneurship Development (YEN-REFINED), Yenepoya (Deemed to be University), Mangalore, India; Lahore Medical and Dental College, PAKISTAN

## Abstract

Type 2 Diabetes Mellitus (T2DM) remains a global health challenge, with increasing prevalence in the Middle East, especially in Qatar. The Primary Health Care Corporation (PHCC) is the main provider of diabetes care in the country. Systematic evaluation through clinical audits provides a structured approach to identify gaps and improve clinical practices. This study aims to evaluate the effectiveness of clinical audit-based interventions in enhancing the management and documentation of T2DM in PHCC settings in Qatar. This quasi-experimental study will be conducted in three phases: pre-intervention, intervention, and post-intervention. In the pre-intervention phase, a cross-sectional situational analysis will be conducted across 31 PHCC health centers by reviewing 450 randomly selected patient records of adults diagnosed with T2DM. A baseline analysis will identify gaps in adherence to evidence-based diabetes care guidelines. The intervention phase will introduce specific quality enhancement strategies, which will encompass staff training, the distribution of guidelines, the engagement of audit champions, and various additional initiatives. Post-intervention, another 450 records will be audited to assess improvement. Additionally, 60 patients will be surveyed via telephone before and after the intervention to assess perceptions of care. The ICAE-DM CARE study aims to evaluate enhancements in clinical documentation, evaluation, and assessment of co-morbidities among patients with diabetes, alongside screening practices, adherence to evidence-based management protocols, and patient satisfaction levels. The findings will facilitate the institutionalization of audit-driven quality improvement initiatives within primary care settings. By providing robust evidence for integrating clinical audits into routine diabetes management, the study seeks to improve patient outcomes and support the objectives outlined in Qatar’s National Health Strategy.

## Introduction

Clinical audit is a well-established process for healthcare quality improvement and patient safety, particularly within hospital environments. It involves systematically reviewing care against explicit criteria, identifying gaps or areas for improvement, and implementing targeted changes to enhance patient outcomes [1]. The process offers numerous benefits, including improved patient care, demonstration of adherence to best practices, more efficient use of resources, increased patient satisfaction, professional development, and support for evidence-based practices [2]. Clinical audits can validate adherence to best practices and pinpoint areas for improvement, promoting transparency and continuous quality enhancement. [1,2].

The worldwide rise in type 2 diabetes mellitus (T2DM) is particularly alarming, with the highest prevalence rates recorded in the Middle East and North Africa at 9.3%, and Qatar exhibiting one of the most significant rates [3]. Projections indicate that by 2050, over 1.31 billion individuals will be diagnosed with diabetes, with prevalence rates surpassing 10%, especially in the Middle East and North Africa, where it is expected to reach 16.8%. This scenario highlights the urgent need for proficient management of diabetes within primary healthcare settings [3].

The application of clinical audits in managing chronic diseases such as type 2 diabetes has shown promising outcomes. For example, interventional audits have led to better management of glycemic levels and blood pressure, along with increased patient satisfaction [4]. Similarly, audits conducted within primary care settings have demonstrated significant improvements in guideline adherence and the appropriateness of diabetes screening and care [5,6]. These initiatives underscore the potential of clinical audits to identify deficiencies, guide targeted interventions, and promote sustained improvements in chronic disease management. In some cases, audits have prompted the development of specialized clinics and longer follow-up visits to ensure ongoing patient care and optimal outcomes [7,8].

Despite these favorable outcomes, there is a notable deficiency in studies investigating the repercussions of an entire audit cycle, particularly regarding chronic disease care, on clinical outcomes and documentation practices. The success of clinical audits largely depends on effectively implementing recommendations in healthcare settings [9]. However, organizational barriers such as limited collaboration between clinicians and management, unclear lines of authority, and differing perspectives can hinder progress. Overcoming these challenges requires fostering a collaborative environment and establishing clear accountability [10].

This study seeks to address a significant gap in literature by evaluating the impact of a systematic clinical audit process on the management and documentation of diabetes within primary care environments. Employing a framework that encompasses both pre- and post-intervention phases in alignment with the ICAE-DM CARE study protocol, this study will evaluate current practices, introduce targeted improvements, and examine the resulting outcomes. The aim is to determine the efficacy of clinical audits in enhancing diabetes care by analyzing variations in diabetes care process indicators, outcome indicators, and related documentation. The results are expected to provide critical insights into the contribution of systematic audit cycles to ongoing quality improvement initiatives. Since 2012, Qatar’s Primary Health Care Corporation (PHCC) has recognized clinical audits as an essential mechanism for ensuring clinical effectiveness, averaging 20–25 audits each year. However, the precise influence of these audits on the management of chronic diseases remains inadequately investigated. This study aims to evaluate the efficacy of clinical audits in improving diabetes care and documentation in accordance with PHCC standards, thereby offering substantial evidence regarding the importance of systematic review processes in enhancing chronic disease management. The study’s methodology, which includes pre- and post-analysis, interventions, and other components, is designed to be replicable across various primary healthcare settings.

## Methodology

### Study design and population

This study employs a mixed-methods approach, comprising a quasi-experimental design for cross-sectional data analysis and a cohort design for patient satisfaction surveys. The research will be conducted over a two-year period, from July 2025 to July 2027. Data will be extracted from the Electronic Medical Records (EMR) system of the Primary Health Care Corporation (PHCC), focusing on patients diagnosed with type 2 diabetes mellitus.

### Study settings

All 31 PHCC health centers providing diabetes care will be included. Data will be collected from stored electronic medical records and direct input from service users. The review of clinical guidelines will be conducted via online access to the PHCC intranet. Additionally, medical records will be reviewed through the Clinical Information System (CIS), and patient satisfaction will be assessed through structured telephone interviews.

### Study population

The study population includes adult patients (≥ 18 years) who are non-pregnant and have attended PHCCs for the management of type 2 diabetes within the past two years. Patients can be newly diagnosed or on follow-up.

**Inclusion Criteria:**

- Age ≥ 18 years
- Confirmed diagnosis of type 2 diabetes mellitus, verified in the problem list or current diagnosis documentation.
- At least two clinical encounters at PHCCs within the last two years
- Ability to communicate verbally via telephone and provide informed verbal consent

**Exclusion Criteria:**

- Pregnant women
- Patients with encounters solely for medication refills without additional clinical data
- Patients below 18 years of age
- Patients unable to communicate by phone or unable to provide verbal consent

### Data collection procedures

Data variables pertinent to the study objectives will be retrieved from the PHCC EMR system, including demographic information, diagnostic details, clinical encounters, and healthcare utilization patterns.

### Telephonic interviews

For qualitative insights and patient satisfaction assessment, eligible patients will be contacted via telephone. The telephonic interview will follow the same inclusion and exclusion criteria, emphasizing the ability to communicate by phone and give verbal consent. Interviews will be conducted by trained personnel, ensuring confidentiality and adherence to ethical standards. Verbal informed consent will be obtained prior to interviews.

This methodological framework allows for comprehensive analysis of clinical data and patient perceptions, facilitating a robust assessment of diabetes management within the primary care setting.

### Sample selection

A stratified simple random sampling method will be employed to select representative samples for both the cross-sectional and cohort components of the study. For the cross-sectional analysis, a total of 450 patient records will be randomly selected from the medical records department across 31 health centers. This sample size was calculated based on a 5% margin of error, a 95% confidence interval, and an assumed response distribution of 50%, with an additional allowance for potential non-responses. The selection will take place at two different time points: baseline and post-intervention, with 450 cases reviewed at each stage to facilitate a comparative analysis of diabetes-related encounters over the preceding three months.

For the patient satisfaction component, a subset of 60 patients will be randomly selected at each time baseline and post-intervention—from the larger cohort. These patients will be contacted by telephone to complete satisfaction surveys, offering qualitative insights into their perceptions of care. The randomization process will be independently applied at both time points to ensure comparability and minimize selection bias. A sample size of 60 patients per time point is considered sufficient to balance feasibility with the need for meaningful analysis of patient satisfaction trends. This approach ensures the robustness and reliability of findings and supports a comprehensive evaluation of both clinical outcomes and patient perspectives throughout the intervention period.

### Medical record review

The medical record review will be conducted by experienced clinical auditors trained in assessing adherence to evidence-based diabetes management guidelines. Data will be systematically recorded using a customized Excel spreadsheet developed specifically for this study, containing all predefined audit criteria.

The review will focus on newly diagnosed type 2 diabetes mellitus (T2DM) patients, particularly those with an HbA1c value ≥6%, to assess whether appropriate clinical services and interventions were delivered in line with guideline recommendations.

The collected data will allow a comprehensive evaluation of healthcare provider adherence, identifying gaps in clinical care and informing quality improvement initiatives across PHCC settings. *(see* Table 1 *for data collection variables).*

**Table 1:** Clinical Audit Criteria and Variables.

Criterion	Description
Criterion 1	ASCVD risk assessment for all adult patients (≥40 years) at diagnosis
Criterion 2.1	Laboratory Investigations: HbA1c, lipid profile (TC, HDL, LDL, TG), serum creatinine and eGFR, liver function (ALT, AST), urine albumin/creatinine ratio, TSH (if dyslipidemia or female >50 years), urine ketones (if glucose >250 mg/dL), serum potassium (if on ACEi/ARB/diuretics)
Criterion 2.2	Initial general foot examination
Criterion 2.3	Referral to eye specialist for initial retinal assessment
Criterion 3	Documentation of HbA1c target goals
Criterion 4	Appropriate treatment initiated or adjusted based on guidelines
Criterion 5	Patient education on diet and physical activity at initial visit
Criterion 6	Referral to dietitian for Medical Nutrition Therapy (MNT)
Criterion 7	Follow-up HbA1c test ordered within 3–6 months from treatment initiation

Source: PHCC clinical practice guideline for the management of Type 2 Diabetes.

### Patient satisfaction survey

A structured telephone-based patient satisfaction survey will be conducted with a sub-sample of patients. The survey is based on key performance indicators (KPIs) aligned with PHCC clinical practice guidelines for diabetes management. This tool was developed and validated by the Department of Clinical Effectiveness to ensure relevance and reliability in measuring patient experience.

Survey data will be recorded using a secure Excel spreadsheet preformatted with all measurement parameters. *(see* Table 2 *for Patient Satisfaction Questionnaire).*

**Table 2:** Patient Satisfaction Survey Indicators.

Question	Indicator
Q1	Did your doctor inquire about and discuss diabetes-related signs and symptoms?
Q2	Did your doctor request any new blood tests or referrals?
Q3	Did your doctor explain how to monitor blood sugar and what to do in case of high/low readings?
Q4	Did your doctor provide advice about diet and physical activity?
Q5	Did your doctor set an HbA1c goal and discuss your treatment plan?
Q6	Overall, are you satisfied with the care provided during your visit?

Source: PHCC clinical practice guideline for the management of Type 2 Diabetes.

### Intervention and follow-up

Following the initial cross-sectional analysis, identified gaps in care quality and patient satisfaction will inform the development of a comprehensive action plan. This plan will be implemented across all participating health centers to address the deficiencies and enhance service delivery. The intervention will be embedded into standard auditing procedures to ensure continuous quality improvement.

**Fig 1:**
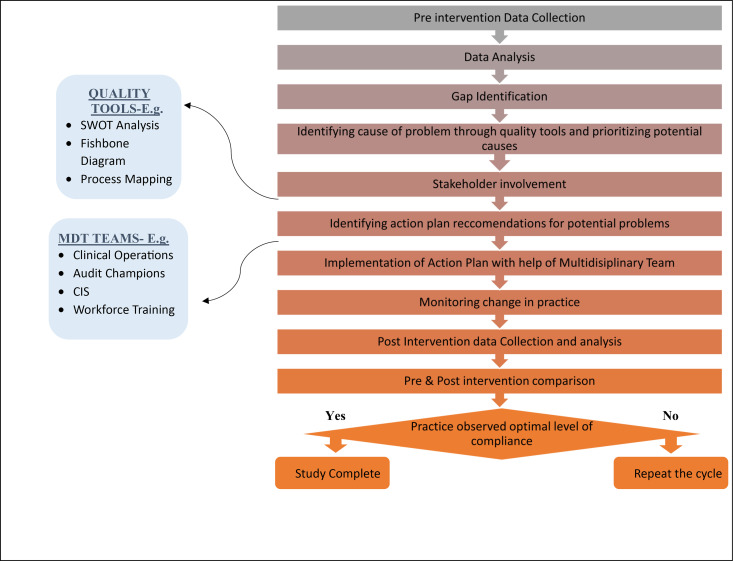
ICAE-DM CARE STUDY flow chart (Author’s own creation).

The intervention phase will last approximately 8–9 months. Afterward, a second round of data collection will be conducted using the same methodology and variables to ensure consistency and enable direct comparison. This approach will support the evaluation of changes in clinical performance and patient experience before and after the intervention *(see* Fig 1 *for the study flow chart).*

### Analytical plan

Data will be extracted from the electronic health record system at two separate points: one time at a point before the intervention and the other time at a point after the implementation of the intervention. Excel will be used to collect and organize the data. PivotTables will be used to determine the frequency of cases across variables. Excel will be further used to produce histograms and other figures.

Study outcome variables will be assessed using independent tests like Chi square or t test to compare between the two groups. For satisfaction, we will do it frequently by percentage P=(F/T) *100.

**Fig 2:**
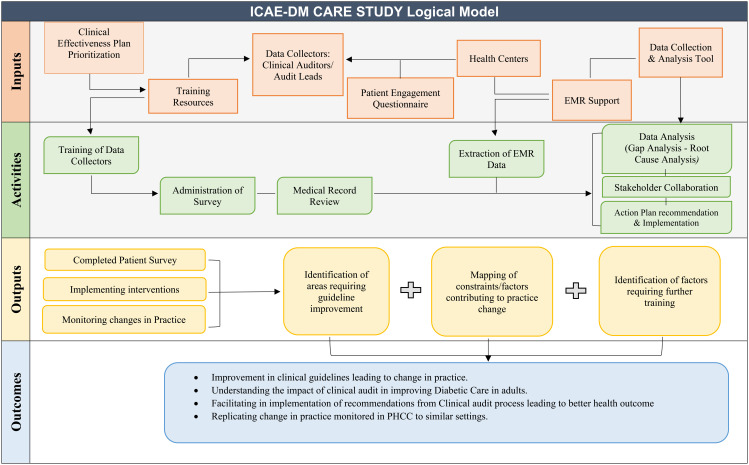
Logical Model (Author’s own creation).

The statistical analysis will be performed using STATA 15.1 (College Station, TX, USA). A Wilcoxon sign rank test will be used to find the mean difference between the pre- and post-assessment. P < 0.05 will be considered statistically significant. A logical model of protocol presented in Fig 2.

### Ethical approval

The Institutional Review Board (IRB) of PHCC (ref no: BUHOOTH-D-24–00059) has reviewed and granted approval for the study on 05/09/2024. Verbal consent will be obtained from participants who are 18 years of age or older for the telephonic survey. The overall planning and execution of the study will be conducted with integrity, in accordance with established ethical principles.

## Discussion

Continuous quality assurance (QA) initiatives are essential for evaluating clinical practices to ensure care is delivered as intended [11]. Diabetes, a chronic condition characterized by elevated blood glucose levels, requires ongoing management to prevent complications and optimize outcomes. Implementing continuous QA efforts is especially critical in both pre-diabetic and diagnosed diabetic populations to sustain effective strategies and enhance patient care [12]. Adherence to established clinical guidelines plays a central role in delaying disease progression and minimizing complications in individuals with type 2 diabetes mellitus (T2DM) [13].

This study was designed to replicate real-world challenges in T2DM management in primary care settings, with the goal of identifying current practice gaps and evaluating the effectiveness of targeted quality improvement initiatives. Through pre- and post-intervention assessments, the study will analyze whether the implemented action plans result in meaningful improvements in care delivery and documentation, or whether unintended consequences emerge. The design facilitates a continuous feedback loop that supports iterative enhancements over time.

The ICAE-DM CARE Study is novel in targeting primary care—the foundational tier where most diabetes management occurs. This setting is particularly relevant in resource-constrained environments. The multi-level intervention strategy combines clinical audits, health provider training, patient engagement, and the use of electronic medical records (EMRs) to facilitate sustainable improvements. Using a quasi-experimental pre-post design, the study integrates both clinical outcomes and patient satisfaction metrics, reflecting a patient-centered approach aligned with global health improvement priorities.

The initial phase of the study involves comprehensive situational analysis through structured chart reviews and telephone-based patient satisfaction surveys. This baseline data allows for a well-rounded understanding of current practices and patients’ experiences.

The findings from this baseline assessment are used to identify deficiencies in clinical practice. Root cause analysis using tools such as fishbone diagrams, process mapping, and SWOT analysis is then conducted to tailor quality improvement (QI) interventions to the context [14,15]. The action plans are implemented collaboratively by a multidisciplinary team, comprising the clinical effectiveness unit, clinical operations, information systems (CIS), and the designated clinical audit champions at each health center.

### The role of clinical audit champions

Clinical audit champions play a key role in bridging the gap between strategic planning and on-the-ground implementation. They are responsible for coordinating local efforts, monitoring compliance with action plans, and reporting progress [16–18]. In this study, clinical audit champions were instrumental in tracking implementation activities at the health center level. They maintained logs and shared periodic updates during six-weekly regional meetings organized by the clinical effectiveness team. These meetings served to track key metrics, troubleshoot challenges, and document improvements or barriers encountered during implementation.

Importantly, audit champions were authorized, in coordination with health center managers, to implement changes aligned with the approved QI plan. Changes implemented included improvements in documentation practices (e.g., timely HbA1c goal setting, foot exam documentation), adherence to referral protocols, and patient counseling activities. These modifications were monitored using structured tracking forms and validated during follow-up audits, providing clear evidence of implementation progress.

### Structured follow-up and monitoring

A formal follow-up structure was embedded into the protocol, where clinical audit champions and regional clinical effectiveness focal points conducted collaborative reviews every six weeks. These recurring sessions allowed champions to present updates, share challenges, and receive guidance. This structured follow-up ensured accountability and supported the continuity and sustainability of changes implemented during the intervention phase.

### Evaluation of audit impact

The effectiveness of the clinical audit was assessed across two domains: clinical care delivery and documentation practices. Improvements in risk assessment documentation, appropriate referrals, lab test completion, and patient education were key outcome indicators. In centers where outcomes were not achieved, qualitative insights revealed constraints such as staff turnover, limited training continuity, and EMR documentation challenges as contributing factors.

The post-intervention phase, scheduled 8–9 months after implementation, mirrors the baseline assessment in design and sample size. The use of consistent tools—open chart reviews and structured surveys ensures comparability between the two data points.

Final comparative analysis will assess the extent of change in care quality indicators and documentation standards following the audit. Where meaningful improvements are found, the results will serve as a basis for scaling similar QI strategies in other primary care settings. Where improvements are minimal, the study will inform refinements in implementation strategies, emphasizing adaptability in quality improvement processes.

This protocol offers a replicable, resource-sensitive model of continuous quality improvement in chronic disease care. Its structured, scalable methodology—rooted in evidence, stakeholder involvement, and iterative evaluation—contributes meaningfully to advancing primary care practices globally.

## Conclusion

This comprehensive study underscores the pivotal role of clinical audits in enhancing the quality of diabetes care within primary healthcare settings. By systematically assessing current practices, identifying care gaps, and implementing targeted multidisciplinary interventions, the ICAE-DM CARE study aims to demonstrate measurable improvements in clinical management, documentation, and patient satisfaction among adults with T2DM. The pre- and post-intervention design provides robust evidence on the effectiveness of structured quality improvement initiatives, highlighting their potential to foster sustainable changes and elevate standards of care. Findings from this study will offer valuable insights into the integration of audit cycles into routine practice, emphasizing the importance of collaborative efforts, continuous monitoring, and data-driven strategies. Ultimately, this approach can serve as a scalable model for primary care facilities worldwide seeking to optimize chronic disease management and improve patient outcomes through systematic quality assurance processes.
